# Supplementary material for: Presence of myxoid stromal change and fibrotic focus in pathological examination are prognostic factors of triple-negative breast cancer: Results from a retrospective single-center study
Source: PLoS One. 2021 Feb 11;16(2):e0245725. doi: 10.1371/journal.pone.0245725 (PMC7877644; doi:10.1371/journal.pone.0245725)
Supplement: S1 Dataset — (PDF) [file pone.0245725.s001.pdf]

No.	Age	menoposal status	BMI	pathological maximum invasive compornent (mm)	positive lymph nodes	pTNM	pStage	histological grading	ly	v	Stromal TILs	Ki-67(Operation: %)	Adjuvant chemotherapy	Fibrotic focus	Myxioid change (Operation)	Myxsoid change (CNB)	relapse	RFS (months)	dead	OS(months)
5	75	-	22.1	15	0	T1cN0M0	I	3	+	-	-	40	+	-	+	+	-	20	-	20
7	31	+	27	12	0	T1cN0M0	I	3	-	-	+	70	+	-	-	-	-	20	-	20
15	41	+	31.2	20	0	T1cN0M0	I	3	-	+	-	40	+	+	-	-	-	35	-	35
16	68	-	32.2	18	0	T1cN0M0	I	3	-	-	+	70	+	-	-	-	-	36	-	36
21	66	-	21.8	22	0	T2N0M0	II A	2	+	-	-	20	+	-	-	-	-	31	-	31
42	83	-	23	18	2	T1cN1M0	II A	3	-	-	-	70	-	-	-	-	-	18	-	18
44	76	-	26	18	0	T1cN0M0	I	2	-	-	+	40	-	-	-	+	-	40	-	40
57	77	-	26.8	6	0	T1bN0M0	I	1	-	-	-	15	+	-	-	-	-	34	+	34
59	65	-	23.3	10	2	T1bN1M0	II A	2	+	-	+	25	+	-	-	+	-	53	-	53
62	77	-	28.9	51	0	T3N0M0	II B	3	+	+	-	80	-	-	-	-	+	11	-	23
63	68	-	24.7	20	0	T1cN0M0	I	2	+	-	-	45	-	-	-	-	-	60	-	60
65	62	-	27.6	28	0	T2N0M0	II A	2	+	-	+	38	+	-	-	+	-	60	-	60
66	74	-	21.7	35	9	T2N2M0	III A	2	+	+	+	50	-	-	-	+	+	18	+	36
67	54	-	21.1	22	0	T2N0M0	II A	2	+	+	-	15	+	-	+	-	-	60	-	60
70	80	-	20.4	12	0	T1bN0M0	I	2	+	+	-	20	-	-	-	+	-	60	-	60
72	93	-	16.2	25	2	T2N1M0	II B	2	+	+	-	40	-	-	-	+	-	60	-	60
73	60	-	25.5	42	0	T2N0M0	II A	2	+	+	+	80	+	-	-	-	-	60	-	60
76	87	-	23.5	55	not tested	T4a cN0M0	III B	3	+	+	-	40	-	+	+	+	-	22	+	29
79	73	-	27.4	38	0	T2N0M0	II A	3	+	+	-	55	unknown	+	-	-	-	44	-	44
81	84	-	24.7	40	0	T2N0M0	II A	3	+	+	-	80	-	+	-	+	-	33	-	60
85	45	unknown	28.9	20	0	T1cN0M0	I	3	+	+	-	70	+	-	-	not available	-	11	-	11
86	67	-	19.9	20	0	T1cN0M0	I	3	+	+	-	50	+	-	-	+	-	60	-	60
87	71	-	20.9	16	1	T1cN1M0	II A	2	+	-	-	70	-	+	-	-	+	9	+	18
88	70	-	23.7	12	0	T1cN0M0	I	2	+	-	+	28	-	-	-	-	-	60	-	60
89	60	-	26.5	13	not tested	T1c cN0M0	I	2	+	-	-	80	-	+	-	+	-	60	-	60
90	88	-	21.1	40	2	T2N1M0	II B	2	+	+	-	50	-	+	+	-	+	22	-	23
91	58	-	21.3	20	not tested	T1c cN0M0	I	3	+	+	+	50	+	-	-	-	-	60	-	60
93	44	+	19.8	27	3	T2N1M0	II B	3	+	+	-	47	+	-	+	+	-	50	-	50
94	69	-	17.3	54	0	T4bN0M0	III B	2	+	-	-	40	unknown	-	+	-	-	60	-	60
97	70	-	26.7	10	0	T1bN0M0	I	1	+	-	-	25	+	-	-	-	-	60	-	60
99	37	+	17.9	52	2	T3N1M0	III A	3	+	+	-	80	+	+	+	-	+	9	+	16
100	80	-	22.6	21	0	T2N0M0	II A	3	+	+	-	80	+	+	-	+	-	60	-	60
102	61	-	25	40	5	T2N2M0	III A	3	+	+	-	25	+	-	-	-	-	48	-	60
103	37	+	23.5	30	0	T2N0M0	II A	3	+	+	-	40	+	-	-	-	-	52	-	52
110	74	-	19.1	25	0	T2N0M0	II A	2	+	+	+	40	+	-	-	-	-	60	-	60
111	78	-	25.7	40	3	T2N1M0	II B	3	+	+	-	49	+	+	-	-	-	60	-	60
118	89	-	20.2	35	not tested	T2 cN0M0	II A	3	+	+	-	5	-	+	+	+	-	46	-	46
119	67	-	27.1	7	not tested	T1b cN0M0	I	2	+	-	-	60	+	-	-	-	-	60	-	60
125	60	-	22.5	6	not tested	T1b cN0M0	I	2	-	-	-	Not evaluated	-	-	-	+	-	60	-	60
127	55	-	27.3	50	0	T2N0M0	II A	3	+	+	-	15	unknown	+	+	+	-	60	-	60
129	44	+	21	30	not tested	T2 cN0M0	II A	3	+	-	+	30	+	-	-	+	-	60	-	60
131	36	+	19.5	13	0	T1cN0M0	I	2	+	-	-	70	+	-	-	-	-	60	-	60
138	62	-	20.2	26	0	T2N0M0	II A	2	+	+	+	10	+	-	-	-	-	60	-	60
140	67	-	18.8	25	0	T2N0M0	II A	2	+	+	-	10	+	-	-	-	-	60	-	60
141	81	-	22.8	18	2	T1cN1M0	II A	2	+	+	-	15	+	+	-	-	-	60	-	60
143	82	-	18.3	12	not tested	T1c cN0M0	I	3	+	+	-	30	-	+	+	not available	-	31	-	31
144	74	-	25.3	23	not tested	T2 cN0M0	II A	2	+	-	-	70	-	+	-	not available	-	60	-	60
145	62	-	31.3	2	not tested	T1a cN0M0	I	2	-	-	+	Not evaluated	-	-	-	not available	-	60	-	60
147	80	-	23.1	40	1	T4bN1M0	III B	3	+	+	-	30	-	+	-	not available	-	56	+	56
148	64	-	25.2	35	4	T2N2M0	III A	2	+	+	-	15	+	+	+	not available	+	40	-	60
149	69	-	27.8	25	0	T2N0M0	II A	3	+	+	+	25	+	-	-	not available	-	60	-	60
151	79	-	23.4	22	not tested	T2 cN0M0	II A	3	+	+	-	50	-	+	+	not available	-	60	+	60
152	54	-	27.3	18	0	T1cN0M0	I	3	+	+	-	60	+	+	+	not available	-	60	-	60
153	58	-	21.9	20	not tested	T1c cN0M0	I	3	+	+	-	15	+	+	+	not available	+	35	+	42
156	50	-	22.5	20	18	T1cN3M0	III C	3	+	+	+	70	-	-	-	not available	+	11	+	41
157	41	+	20.6	17	not tested	T1c cN0M0	I	3	+	+	+	60	+	-	-	not available	-	60	-	60
159	58	-	24	2	0	T1aN0M0	I	3	+	-	+	Not evaluated	+	-	-	not available	-	48	-	48
161	52	-	24	20	0	T1cN0M0	I	3	+	+	+	80	+	-	-	not available	-	60	-	60
162	45	+	16.5	25	0	T2N0M0	II A	3	+	+	-	Not evaluated	-	+	+	not available	+	15	+	57
163	71	-	20.4	12	not tested	T1c cN0M0	I	2	+	-	-	60	-	-	+	not available	-	53	-	53
165	69	-	26.5	7	not tested	T1b cN0M0	I	2	+	-	-	80	-	-	-	not available	-	60	-	60
168	81	-	19.6	40	0	T2N0M0	II A	2	-	-	+	15	-	-	-	not available	-	16	-	16
