# Supplementary material for: Presence of myxoid stromal change and fibrotic focus in pathological examination are prognostic factors of triple-negative breast cancer: Results from a retrospective single-center study
Source: PLoS One. 2021 Feb 11;16(2):e0245725. doi: 10.1371/journal.pone.0245725 (PMC7877644; doi:10.1371/journal.pone.0245725)
Supplement: Attachment — Submitted filename: Response_to_Reviewers.docx [file pone.0245725.s002.docx]

November 30, 2020

Dr. Joerg Heber

Editor-in-Chief

PLoS ONE

Resubmission - Manuscript ID: PONE-D-20-23411

Dear Dr. Heber:

I would like to resubmit an article for publication in *PLoS ONE*, titled “Presence of myxoid stromal change and fibrotic focus in pathological examination are prognostic factors of triple-negative breast cancer: Results from a retrospective single-center study.”

Your comments, as well as those of the reviewers, were highly insightful and enabled us to greatly improve the quality of our manuscript. In the following pages, I have provided our point-by-point response to all comments and have highlighted the revised portions of the manuscript.

I hope that you will find our revised manuscript suitable for publication in *PLoS ONE*. I look forward to hearing from you at your earliest convenience.

Sincerely,

Mitsuaki Ishida

Kansai Medical University

2-5-1, Shinmachi, Hirakata City

Osaka, 573-1010, Japan

Tel: +81-72-804-2794

Fax: +81-72-804-2794

E-mail: ishidamt@hirakata.kmu.ac.jp

**Response to the comments of Reviewer #1**

Thank you very much for reviewing our manuscript. We appreciate your constructive comments. We have made the following revisions in response to the issues raised by you.

1. The authors analyzed only a cohort of 62 TNBC patients. Please add a validation cohort.

**Response**: This study cohort included a relatively small number of patients with triple-negative breast cancer (TNBC), as mentioned in the Discussion section. As you have mentioned, a validation cohort is required to clarify the results of the present study. However, at this point, we are unable to validate the results, and we aim to undertake an international validation cohort study to clarify the usefulness of myxoid change and fibrotic focus in TNBC in the near feature.

2. As reported in Table 1, 56.5% of patients received adjuvant chemotherapy and 38.7% did not received chemotherapy. The analysis of OS and RFS for all stromal prognostic factors considered should be performed considering separately patients who received/not received treatment. Please add these analyses.

**Response**: As you have suggested, we have additional analyses of OS and RFS in TNBC patients with or without adjuvant chemotherapy. We added the results of these analyses in the Results section and showed the survival curves in Figs. 4 and 5 (p19-22). Moreover, we added the following statement regarding the results in the Discussion section.

“*Association between myxoid change, FF, and a combination of myxoid change and FF and survival in patients with adjuvant chemotherapy*

Consequently, we performed survival analyses in patients with or without adjuvant chemotherapy. Myxoid change was observed in 7 of 34 patients (20.6%) with adjuvant chemotherapy. Fig. 4a shows the OS and RFS curves of myxoid change-positive and -negative patients with adjuvant chemotherapy. The presence of myxoid change significantly correlated with poor RFS (*p=0.002*) but not with OS (*p=0.080*). FF was observed in 8 of 34 patients (23.5%) with adjuvant chemotherapy. Fig. 4b shows the OS and RFS curves of FF-positive and -negative patients with adjuvant chemotherapy. The presence of FF significantly correlated with poor RFS (*p=0.004*) but not with OS (*p=0.13*). Both myxoid change and FF were observed in 4 of 34 patients (11.8%) with adjuvant chemotherapy. Fig. 4c shows the OS and RFS curves of myxoid change- and FF-positive vs myxoid change-and/or FF-negative patients with adjuvant chemotherapy. The presence of a combination of myxoid change and FF significantly correlated with poor OS (*p=0.007*) and RFS (*p<0.001*).

**Fig. 4** Survival analyses for myxoid change, fibrotic focus (FF), and a combination of myxoid change and FF in patients with adjuvant chemotherapy.

(a) Kaplan–Meier curves for the overall survival (OS) and relapse-free survival (RFS) of patients with adjuvant chemotherapy. OS (left) and RFS (right) curves among myxoid change-positive (red line) and myxoid change-negative (blue line) patients. (b) OS (left) and RFS (right) curves among FF-positive (red line) and FF-negative (blue line) patients. (c) Overall survival (OS) (left) and relapse-free survival (RFS) (right) curves in myxoid change and FF-positive (red line) and myxoid change- and/or FF-negative (blue line) patients.

*Association between myxoid change, FF, and a combination of myxoid change and FF and survival in patients without adjuvant chemotherapy*

Myxoid change was observed in 7 of 25 patients (28.0%) without adjuvant chemotherapy. Fig. 5a shows the OS and RFS curves of myxoid change-positive and -negative patients without adjuvant chemotherapy. The presence of myxoid change significantly correlated with poor OS (*p=0.030*) but not with poor RFS (*p=0.069*). FF was observed in 11 of 25 patients (44.0%) without adjuvant chemotherapy. Fig. 5b shows the OS and RFS curves of FF-positive and -negative patients without chemotherapy. The presence of FF did not significantly correlate with OS (*p=0.068*) and FRS (*p=0.080*). Both myxoid change and FF were observed in 6 of 25 patients (24.0%) without adjuvant chemotherapy. Fig. 5c shows the OS and RFS curves of myxoid change- and FF-positive vs myxoid change-and/or FF-negative patients without adjuvant chemotherapy. The presence of a combination of myxoid change and FF significantly correlated with poor OS (*p=0.027*) but not with RFS (*p=0.68*).

**Fig. 5** Survival analyses for myxoid change, fibrotic focus (FF), and a combination of myxoid change and fibrotic focus in patients without adjuvant chemotherapy.

(a) Kaplan–Meier curves for the overall survival (OS) and relapse-free survival (RFS) of patients without adjuvant chemotherapy. OS (left) and RFS (right) curves among myxoid change-positive (red line) and myxoid change-negative (blue line) patients. (b) OS (left) and RFS (right) curves among FF-positive (red line) and FF-negative (blue line) patients. (c) Overall survival (OS) (left) and relapse-free survival (RFS) (right) curves in myxoid change and FF-positive (red line) and myxoid change- and/or FF-negative (blue line) patients.” (page 19-22)

“Moreover, for the first time, the present study analyzed the survival significance of the presence of myxoid change and/or FF in TNBC patients with or without adjuvant chemotherapy. According to the results of the present study, the presence of myxoid change, FF, and the combination of myxoid change and FF were significant poor prognostic factors for RFS in patients with adjuvant chemotherapy. However, this trend was not noted in patients without adjuvant chemotherapy. Besides, myxoid change-, FF-, and myxoid change and FF-positive patients showed similar survival curves regardless of the presence of adjuvant chemotherapy; however, myxoid change-, FF-, and myxoid change and/or FF-negative patients without adjuvant chemotherapy showed poorer survival curves compared to those of patients with adjuvant chemotherapy. Accordingly, adjuvant chemotherapy might not be efficient in improving outcomes in myxoid change- and/or FF-positive TNBC patients.” (page 25)

3. In figures 1a and 2a the authors report only a representative image at large magnification for myxoid change and fibrotic focus respectively. Please add also images at lower magnification and images of tumor sample negative for myxoid change and fibrotic focus.

**Response**: As you have suggested, we have added the images with a lower magnification of myxoid change (Figs. 1a, ab) or fibrotic focus (Figs. 2a, 2b). We have also added the figures without myxoid change (Fig. 1c) or fibrotic focus (Fig. 2c).

4. Correlation analysis with clinicopathological factors, reported in Table 2 was done only considering patients with both mixoid change and fibrotic focus together. Please add correlation analysis considering separately the two stromal characteristics.

**Response**: As you have suggested, we have performed additional analyses of the association of myxoid change or fibrotic focus with clinicopathological characteristics, separately. We have illustrated these results in Table 2 and Table 3. The results were fundamentally the same as those of myxoid change and fibrotic focus (Table 4).

5. In the introduction section the authors should better support sentences at lines 62-65 with references from literature

**Response**: As you have suggested, we have added reference 5.

6. Table 1: tumor size is considered as a continuous variable. Could the authors indicate tumor size as they do in Table 2 and 3 for correlation and multivariate analyzes?

**Response**: We agree with you that tumor size is a continuous variable. In this cohort, the median tumor size was 20 mm. Notably, TNM classification of the breast cancer classified T1 as 2cm or less, and T2 and T3 as more than 2 cm. Thus, we classified the tumor size as ≦2 cm or >2 cm in this analysis.

7. As regards the analysis of TILs, the paper the authors refer to, considers also “intermediate TILs as 10%

**Response**: As you have suggested, TIL is classified as low (0-10%), intermediate (11-59%), and high (more than 60%) according to Reference 12. Thus, in this study, we classified TIL as low/intermediate (0-59%) and high (more than 60%).

**Response to the comments of Reviewer #2**

Thank you very much for reviewing our manuscript. We appreciate your constructive comments. We have made the following revisions in response to the issues raised by you.

1. Although there are two cited papers regarding the criteria to detect myxoid change it would be useful to clearly explain the characteristics taking into account for the analysis.

**Response**: As you have suggested, we have added a statement regarding the histopathological characteristics of myxoid change in the methods section.

“Myxoid change was histopathologically characterized by the presence of amorphous extracellular substances, which comprise an amphophilic or slightly basophilic material within the fibrous stroma” (page 6)

2. There is any score to classify positive and negative tissues (%, number of cells, area)?, what was to cutoff to consider a positive tissue?

**Response**: In the present study, we classified tissues as positive when typical myxoid change was present around the tumor according to colorectal cancer criteria (Ref. 6,7), and no cutoff value was present. As you have suggested, definitive assessable and reproductive criteria for myxoid change in breast cancer are needed (Discussion, page 26).

3. Please include at least two more representative pictures of myxoid changes and FF, and clearly indicate by arrows the area and changes of interest. Please also include images from patients in which the myxoid changes or FF were not observed.

**Response**: As you have suggested, we have added images with a lower magnification of myxoid change (Figs. 1a, ab) or fibrotic focus (Figs. 2a, 2b). We have also added the figures without myxoid change (Fig. 1c) or fibrotic focus (Fig. 2c).

4. On table 2 why positive patients to FF or Myxoid change were grouped with negative patients?. I would group the tumors as following to get a better idea of the independent and coordinate changes: 1) Myxoid change and fibrotic focus-positive (N=11) 2) Myxoid change positive-only (N=5) 3) fibrotic focus-positive (N=10) and 4) Myxoid change and fibrotic focus-positive negative (N=36).

**Response**: We have performed additional analyses regarding the association of myxoid change or fibrotic focus with clinicopathological characteristics, separately. We have illustrated these results in Table 2 and Table 3. The results were fundamentally the same as those of myxoid change/fibrotic focus (Table 4). We think that analyses of four groups (myxoid change and/or fibrotic focus) might be difficult because there were only 5 patients in the myxoid change-positive/fibrotic focus-negative group.

5. As previously mentioned on table 3 it is necessary to show multivariate analysis of stroma changes occurring independently and in co-occurrence, to get an idea of how this phenomena impact tumor biology alone or in combination.

**Response**: As you have suggested, we have performed multivariate analyses of relapse-free survival of myxoid change or fibrotic focus, separately. We have added the results as follows.

“In addition, a multivariate analysis of RFS performed as a separate factor of myxoid change or FF showed that both myxoid change (HR 1.78; 95% CI 0.13-23.7; *p=0.66*) and FF (HR 5.18; 95% CI 0.34-79.7; *p=0.24*) were not independent poor prognostic factors.” (page 18)

6. Although this is one of the first efforts to associate the presence myxoid changes and FF with clinical outcomes and patients features on TNBC, unfortunately by the limited number of included tumors there is not an optimal statistical strategy that can be robustly applied and the conclusion are not strongly supported, thus I encourage the authors to include some independent cohorts to confirm some of their findings. Since hematoxylin and eosin is the only necessary test to evaluate stroma reactions, I will suggest including some triple negative TCGA tumors that can be evaluated from <https://cancer.digitalslidearchive.org/#!/CDSA/brca> and clinical and TILS information is available.

**Response**: We appreciate your kind suggestion. This study cohort included a relatively small number of patients with triple-negative breast cancer (TNBC), as we have acknowledged in the Discussion section. As you mentioned, a validation cohort is required to clarify the results of the present study. However, at this point, we are unable to validate the results, and we aim to undertake an international validation cohort study to clarify the usefulness of myxoid change and fibrotic focus in TNBC in the near feature.

In the present study, the presence of myxoid change or fibrotic focus was examined using surgically resected specimens (more than 2 slices in all cases). While the tool you have suggested is very useful, we believe it is not suitable for this study since only one slice was available in each case.

7. Since most of the triple negative tumors are now being treated in neoadjuvant therapy, it is possible to examine this stroma reactions from biopsy tissues?. The authors described that they discard an important set of tumoral samples from their original cohort due to neoadjuvant treatment. So 1) It is possible to compare the histopathological assessment of the surgical analyzed tissues, included in this study, with their matched diagnosis biopsies to define how much a biopsy tissue can recapitulate what is detected in a larger tissue (surgical sample) 2) it is possible to include the analysis of this excluded patients based on their biopsy tissue? Please discuss this point.

**Response**: As you have suggested, we have examined the usefulness of analysis of the matched pre-operative biopsy specimens. We have added the results.

“Moreover, we also evaluated the presence of myxoid change and FF using the matched pre-operative biopsy specimens.” (page 6-7)

“*Comparison of the presence of myxoid change between pre-operative biopsy specimens and surgically-resected specimens*

Pre-operative biopsy specimens were available in 44 patients (71%) in this cohort. Of the 11 myxoid change-positive patients diagnosed via surgically resected specimens, 9 patients were positive for the myxoid change via biopsy specimens. Besides, of the 33 myxoid change-negative patients diagnosed via surgically resected specimens, 25 patients were negative for myxoid change via biopsy specimens. There was a moderate correlation between biopsy and surgically resected specimens regarding the presence of myxoid change (Kappa statistics: 0.487, *p=0.001*). FF was noted only in 5 biopsy specimens; thus, statistical analysis was not performed.” (page 21-22)

“Additionally, the present study demonstrated a moderate correlation between pre-operative biopsy and surgically resected specimens regarding the presence of myxoid change. Thus, evaluation of the presence of myxoid change in the biopsy specimens might provide prognostic information in patients with TNBC because many patients with TNBC are currently treated with neoadjuvant chemotherapy.” (page 24-25)

8. Since a Fisher test was computed to examine the significance of the association between two kinds of classification the most accurate term for table 2 and methods section is association instead of correlation.

Response: As you have suggested, we have changed “correlation” to “association”.

“The association between groups was evaluated using Fisher’s exact test for categorical variables” (page 7)
